# Intermixing the *OPN1LW* and *OPN1MW* Genes Disrupts the Exonic Splicing Code Causing an Array of Vision Disorders

**DOI:** 10.3390/genes12081180

**Published:** 2021-07-29

**Authors:** Maureen Neitz, Jay Neitz

**Affiliations:** Department of Ophthalmology and Vision Science Center, University of Washington, Seattle, WA 98109, USA; jneitz@uw.edu

**Keywords:** colorblindness, color vision, myopia, cone photopigment, exon skipping, X-linked cone dysfunction

## Abstract

Light absorption by photopigment molecules expressed in the photoreceptors in the retina is the first step in seeing. Two types of photoreceptors in the human retina are responsible for image formation: rods, and cones. Except at very low light levels when rods are active, all vision is based on cones. Cones mediate high acuity vision and color vision. Furthermore, they are critically important in the visual feedback mechanism that regulates refractive development of the eye during childhood. The human retina contains a mosaic of three cone types, short-wavelength (S), long-wavelength (L), and middle-wavelength (M) sensitive; however, the vast majority (~94%) are L and M cones. The *OPN1LW* and *OPN1MW* genes, located on the X-chromosome at Xq28, encode the protein component of the light-sensitive photopigments expressed in the L and M cones. Diverse haplotypes of exon 3 of the *OPN1LW* and *OPN1MW* genes arose thru unequal recombination mechanisms that have intermixed the genes. A subset of the haplotypes causes exon 3- skipping during pre-messenger RNA splicing and are associated with vision disorders. Here, we review the mechanism by which splicing defects in these genes cause vision disorders.

## 1. Introduction

Exons comprise overlaid splicing and protein codes. Exonic splicing enhancers and silencers (ESEs and ESSs) were initially discovered in alternatively spliced exons but are now known to control the splicing of constitutive exons [1,2,3,4,5,6,7]. The long-wavelength sensitive (L) and middle wavelength sensitive (M) opsin genes (*OPN1LW* and *OPN1MW*, respectively) of Old World nonhuman primates have stereotyped differences [8] that presumably were shaped by the dual evolutionary pressures of the superimposed exon splicing and protein codes [9,10]. In contrast to these nonhuman primates [11,12,13], humans show extreme variation in the L and M opsin gene sequences [14,15]. Genetic polymorphisms are usually assumed to be attributable to the pressure of selective advantage driving an increase in the frequency of different variants in the population; however, this is not the case for the L and M opsin genes [8]. The *OPN1LW* and *OPN1MW* genes are arranged in a head-to-tail tandem array on the X-chromosome [16,17]; they share greater than 96% nucleotide sequence identity over their entire ~40 kilobase pair (kb) length [18]. Duplicated genes are prone to unequal homologous recombination and non-reciprocal transfer of genetic information [19]. These recombination mechanisms promote interchange of DNA between duplicated genes simultaneously creating high haplotype diversity and reducing the divergence between duplicates [20]. Sequence analyses of the human genes indicate that the opsin gene array has a history of unequal homologous recombination responsible for producing gene rearrangements that underlie red-green color vision deficiency (CVD) [16]. Recombination mechanisms have contributed to a high mutation rate in which the sequences of the primordial L and M opsin genes have been interchanged [8,14,15,18,21,22].

Among mammals, only primates have trichromatic color vision. Moreover, in humans and Old World nonhuman primates, trichromatic color vision is the norm (commonly referred to as normal color vision) for both males and females [23]. Presumably, the “mutation pressure” associated with a high rate of interchanging the L and M opsin gene sequences is counteracted in nonhuman primates by robust selection pressure against red-green color vision deficiencies. This may explain the absence of L/M interchange variants in nonhuman primates. Selection against colorblindness is relaxed in modern humans who, for example, do not rely on color vision to obtain food. Thus, the ongoing intermixing of the human L and M opsin genes is undoing evolution’s handiwork in generating well-designed photopigment genes in the face of constraints on protein structure and function and splicing signals. The result is that some combinations of single nucleotide polymorphisms (SNPs) that are individually benign are associated with cone photoreceptor dysfunction, and interchange variants are becoming recognized as important causes of vision loss [8,22,24,25,26,27,28,29,30,31,32,33].

The *OPN1LW* and *OPN1MW* genes each have six exons [16]. Exons 1 and 6 vary only rarely [14]. Exon 5 encodes amino acid differences that functionally distinguish L from M photopigments [34], and it differs in a somewhat stereotyped fashion between L and M opsin genes [34,35]. Exons 2, 3, and 4 vary among and between the L and M opsin genes [36]. There are four common SNPs in exon 2, eight in exon 3, and five in exon 4. Of the eight SNPs in exon 3, two are silent (c.453 and c.465), two occur in codon 171 (c.511 and c.513), and together specify an amino acid substitution, and the remaining four (c.457, c.521, c.532 and c.538) each specify an amino acid substitution (Table 1).

Vision disorders have been frequently associated with four interchange variants that have specific combinations of the exon 3 SNPs [24,25,26,27,28,29,30,31,32,33,37]. In the literature, authors have abbreviated the interchange variants using the single letter code to indicate the amino acids specified at each variant position encoded by exon 3. The variant amino acid positions are p.153, p.171, p.174, p.178, and p.180 (see Table 1 and Table 2). The four variants frequently associated with vison disorders are LIAVA (Leucine 153, Isoleucine 171 Alanine 174, Valine 178, Alanine 180), LVAVA (Leucine 153, Valine 171, Alanine 174, Valine 178, Alanine 180), MIAVA (Methionine 153, Isoleucine 171 Alanine 174, Valine 178, Alanine 180), and LIAVS (Leucine 153, Isoleucine 171 Alanine 174, Valine 178, Serine 180). These variants are associated with clinical diagnoses of red-green color vision deficiency [24,26,37], blue cone monochromacy [22,25,27], high grade myopia [32,38], cone dysfunction [22,31], cone dystrophy [22,31], and Bornholm Eye Disease [30,33,39]. Until recently, the mechanism whereby these variant opsin genes cause vision problems had not been clear. Moreover, the diversity of phenotypes associated with “interchange variants” has been challenging to explain.

**Figure 1:**
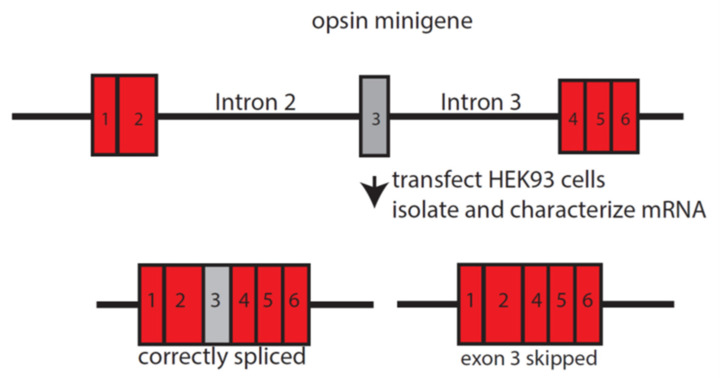
Opsin minigene splicing assay. Top: The *OPN1LW* minigene is *OPN1LW* cDNA with introns 2 and 3 inserted. The introns are labeled, and the numbered red rectangles are the exons that were identical in sequence for all minigenes. The gray rectangle is exon 3, which varied in sequence among the minigenes. The effects of different SNP combinations in exon 3 on splicing were measured by first transfecting HEK293 cells with minigenes. mRNA was isolated from HEK293 cells, and the amount of correctly spliced mRNA was quantified. Data in Table 2 are from reference [22], and the percentage of correctly spliced mRNA was measured using capillary gel electrophoresis.

Recently, studies demonstrated that the LIAVA, LVAVA, MIAVA, and LIAVS haplotypes of the L and M opsin genes are associated with different degrees of exon 3- skipping [29]. Three additional combinations, MVVVA (Methionine 153, Valine 171, Valine 174, Valine 178, Alanine 180), MVAVA (Methionine 153, Valine 171, Alanine 174, Valine 178, Alanine 180), and LIAIA (Leucine 153, Isoleucine 171, Alanine 174, Isoleucine 178, Alanine 180), have also been argued to be disease-causing [22]. Table 2 shows the haplotypes for disease-associated variants, both using the nucleotide information (left side of Table 2) and amino acid information (right side of Table 2). There are two nucleotide haplotypes associated with the LIAVA, MIAVA, and MVVVA variants. The difference between each pair of haplotypes is the silent nucleotide substitution in codon 151 at nucleotide position c.465. The percentages of correctly spliced mRNA observed for these variants appear in the rightmost column in Table 2. The splicing data in Table 2 are from reference [22] and are the results of the minigene splicing assay (described in Figure 1). For haplotypes 1 through 6 and 10 in Table 2, the major splicing isoform observed was missing exon 3 and had exon 2 spliced directly to exon 4, altering the reading frame in codon 138 and introducing a premature translation termination signal at codon 143. For haplotypes 7, 8, and 9, the major splicing isoform observed was the correctly spliced message, but exon 3-skipped mRNA was also observed (Table 2). Thus, one key difference among these disease-associated haplotypes is the relative amount of functional photopigment made in the cones [40].

For haplotype pairs 5/6 and 7/8 (Table 2), the two percentages of correctly spliced mRNA correspond to the two haplotypes that differ a c.465. The first percentage for each pair of haplotypes corresponds to the haplotype with the first nucleotide indicated at position c.465. The second value is the percentage for the second nucleotide indicated at position c.465. Haplotypes 1 and 2 failed to yield any measurable amount of correctly spliced mRNA [22,40], corresponding to zero percent correctly spliced mRNA.

The association between the LIAVA and LVAVA variants and vision disorders was first reported in the literature in 2004 [24,25,26,37]. However, the first published report that these haplotypes cause exon 3-skipping did not appear until 2012 [29]. The literature before 2012 focused on the potential for amino acid substitutions to disrupt photopigment structure and function. Overlooking the potential for nucleotide changes in protein-coding regions to alter splicing is a generalized, widespread problem, which has left a significant gap in our knowledge regarding the underlying mechanism by which nucleotide polymorphisms cause disease [41]. The evidence reviewed here supports the conclusion that exon 3-skipping, not protein sequence variation, is primarily responsible for the vision disorders associated with the LIAVA, LVAVA, MIAVA, and LIAVS variants.

The splicing defects associated with the *OPN1LW* and *OPN1MW* exchange mutants affect cone function, which directly impacts vision. However, one of the most devastating consequences is indirect, being the result of disruption of normal eye growth, which is controlled by signals from the cones that are disturbed by the mutations. Specifically, the LVAVA splicing-defective opsin gene haplotype is associated with both syndromic and non-syndromic myopia caused by excessive axial growth of the eye [22,30,32,33]. The role of cone photoreceptors in controlling eye growth is significant because myopia is a leading cause of blindness globally. Myopia’s prevalence has risen dramatically in recent decades [42,43]. Refractive error is correctable with lenses or surgically, but these treatments do not address the underlying cause of myopia, which is the excessive axial elongation of the eye, nor do they reduce the risk of blindness due to complications secondary to myopia [44]. An estimated 5 billion people (half the world’s population) will be myopic by the year 2050, and 1 billion people (9.8% of the world population) will have high myopia [43,45]. The prevalence of myopia in the U.S. doubled over the last 30 years [42], and the prevalence of myopia worse than -8.00 diopters (D) increased eight-fold [46]. There are at least 160 million young myopes in China and 16 million with pathological myopia (defined as worse than −6.0 D) [45]. A younger age-at-onset of myopia is of particular concern because younger eyes progress more rapidly and achieve higher levels of myopia [47]. The projected global health care burden caused by myopia is staggering [42,45,48,49]. Despite robust evidence that myopia has a large genetic component [43,44,46,50,51,52,53,54,55,56,57], the search for common myopia genes with clinically significant contributions to the disease has failed [56].

Retinal circuitry extracts visual information by comparing signals from adjacent cones [58,59]. Adjacent cones harboring different amounts of functional photopigment will signal the presence of contrast, even when there is no contrast in the image [60]. An inherited form of high myopia linked to Xq28 [59] occurs in males who express an Xq28 cone opsin gene with the LVAVA haplotype and second cone opsin gene with a haplotype exhibiting very little exon 3-skipping [30]. Thus, these high myopes have two types of L/M cones that harbor dramatically different amounts of photopigment. This observation led us to hypothesize that other genetic and environmental factors may produce a similar or more mild abnormality in contrast signaling and underlie the common juvenile-onset form of myopia that is the source of concern for the global burden of myopia. We reasoned that if abnormal contrast signaling is the stimulus for the eye to grow and leads to excessive axial elongation, it should be possible to slow or prevent axial elongation with spectacle lenses that reduce contrast. Such lenses have been developed [61] and are currently in testing for safety and efficacy in slowing the progression of myopia in children (National Clinical Trials.gov study ID NCT03623074).

## 2. LIAVA and LVAVA Haplotypes Affect Vision Primarily through Abnormal Splicing, Not Abnormal Protein Structure or Function

Initial reports that the LIAVA and LVAVA haplotypes disrupt cone function suggested that they did so through defects in protein structure or function [24,25,26]. Genetically modified mice were created to assess the function of the LIAVA and LVAVA pigments independent of the splicing defect [40]. Mice harbored a modified human *OPN1LW* cDNA inserted into the endogenous mouse X-chromosome cone opsin gene locus. A detailed description of the transgene is provided in reference [40] and not reiterated here; however, the critical feature is that the primary transcript from the transgene contained only one intron (intron 1), thereby circumventing the splicing defect caused by the exon 3 haplotype. Until about 16 months of age, the mice with the LIAVA variant did not differ in cone function compared to mice harboring a normal control transgene. However, mice with the LIAVA opsin transgene did differ from the control at 16 months of age, leaving open the possibility that the LIAVA photopigment causes a mild, late-onset harmful effect. In contrast, mice with the LVAVA variant exhibited reduced cone function compared to the control mice at every age examined, consistent with it causing slow cone degeneration.

Most published studies of cone function in human males with the LIAVA or LVAVA cone opsin haplotypes report using a standard clinical photopic electroretinogram (ERG); however, this technique cannot isolate the responses specifically of the L and M cones. Greenwald et al. [40] used a long-flash electroretinogram (ERG) with a custom L/M cone-isolating stimulus [62] to investigate cone function in human males with either the LIAVA or LVAVA opsin haplotype expressed in all of their cones except the S cones. The amplitude of the ERG b-wave corresponded to the expected amount of photopigment based on the opsin gene haplotypes in these subjects [40]. Together, these data support the conclusion that the splicing defect is primarily responsible for the disease phenotypes associated with these haplotypes. The human data were also consistent with a slow degeneration associated with LVAVA but not LIAVA, presumably due to the small amount of mutant photopigment made in cones expressing the LVAVA haplotype.

## 3. The Architecture of the Opsin Gene Array and Gene Order Are Important Determinants of Color Vision Phenotype

**Figure 2:**
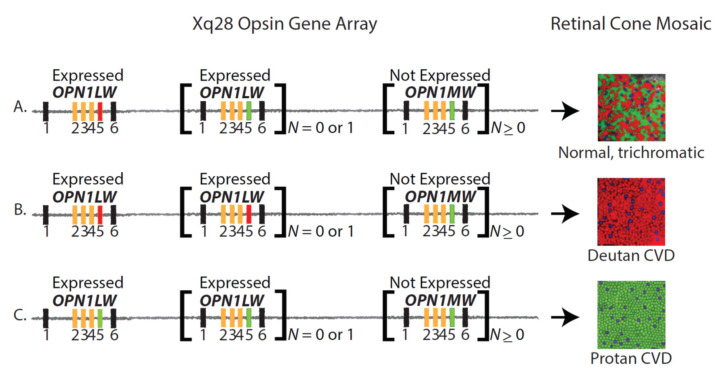
Opsin gene array structure, cone mosaic, and color vision phenotype. The numbered rectangles represent the exons of the *OPN1LW* and *OPN1MW* genes. Black rectangles represent exons 1 and 6. Exons 2, 3, and 4 are polymorphic among and between the *OPN1LW* and *OPN1MW* genes and thus are colored yellow. Exon 5 encodes two amino acid differences that functionally distinguish L from M photopigments [34]; therefore, red rectangles represent exon 5 of *OPN1LW* genes, and green rectangles represent exon 5 of *OPN1LW* genes. (**A**) An opsin gene array with the two expressed positions occupied by *OPN1LW* and *OPN1MW* genes will give rise to a retinal cone mosaic with L cones (red circles), M cones (green circles), and S cones (blue circles) and will confer normal trichromatic color vision. (**B**) An opsin gene array with only one *OPN1LW* gene (second and third genes *N* = 0) or the two expressed positions occupied by *OPN1LW* genes (second gene *N* = 1) gives rise to a retinal mosaic with L cones and S cones, but no M cones, and will underlie deutan CVD. (**C**) An opsin gene array with one *OPN1MW* gene (second and third genes *N* = 0) or with both expressed positions (second gene *N* = 1) occupied by an *OPN1MW* gene will give rise to a retinal mosaic with only S cones and M cones, but no L cones and will underlie protan CVD. Please see references [8,69] for recent reviews of the genetics of normal and defective color vision.

In humans, non-reciprocal recombination mechanisms have generated gene rearrangements that underlie the common inherited color vision deficiencies and copy number variation in people with normal color vision [17,18] and the relatively rare disorder blue cone monochromacy [63]. Normal trichromatic color vision requires short-wavelength (S), long-wavelength (L), and middle-wavelength (M) sensitive cones (commonly referred to as blue, red, and green cones). Figure 2A illustrates the most common arrangement in individuals with normal color vision: an *OPN1LW* gene followed by one or more *OPN1MW* genes [16,18]; however, only the first two genes in the array are expressed [64]. Upstream of the opsin gene array is a locus-specific enhancer termed the locus control region (LCR) [65]. The *OPN1LW* and *OPN1MW* genes share the same LCR; thus, only one gene is expressed at a time in each cone photoreceptor [65,66]. Moreover, epigenetic mechanisms [67] ensure that *OPN1LW* and *OPN1MW* genes are transcribed in separate populations of cone photoreceptors, as required for trichromatic color vision. The choice of which opsin gene to express, the first or second in the array, is stochastic and there are no known molecular distinctions between L and M cones besides the opsin gene they express [68].

### 3.1. Blue Cone Monochromacy (BCM)

Vision in blue cone monochromats is mediated solely by S cones and rod photoreceptors. Blue cone monochromats have poor visual acuity because they have no functional L or M cones. Deleting the LCR is a common cause of blue cone monochromacy because it prevents transcription of all the X-chromosome cone opsin genes [63,70]. Another cause of BCM is the deletion of all but one X-chromosome cone opsin gene and the presence of inactivating mutation in the remaining gene [63,70]. Exon 3-skipping haplotypes of the *OPN1LW* or *OPN1MW* gene also cause blue cone monochromacy, as described in more detail below.

### 3.2. Red-Green Color Vision Deficiency

About 8% of males (1 in 12) and 0.4% of females (1 in 230) are affected by some form of inherited red-green color vision deficiency. Although three cone types, L, M, and S, mediate normal color vision (Figure 2A), two cone types mediate inherited red-green color vision deficiency (Figure 2B,C, respectively). Deutan, derived from Greek for “the second type,” is the term for CVD mediated by S and L cones and is the most common form, accounting for about 6% of males. Protan, derived from Greek for “the first type,” is the term for CVD mediated by S and M cones.

Deutan and protan CVD each come in two forms, dichromatic (deuteranopia and protanopia) and anomalous trichromatic (deuteranomaly and protanomaly). Males have deuteranopia if the first and second genes specify L cone photopigments that are identical in their wavelengths of peak sensitivity or if they have a single gene array that encodes an L pigment (Figure 2B). Males have deuteranomaly if the first and second genes encode L cone photopigments that differ in the wavelength of peak sensitivity (Figure 2B). Males have protanopia if the first and second genes specify M cone photopigments that are identical in their wavelengths of peak sensitivity or if they have a single gene array that encodes an M pigment (Figure 2C). Males have protanomaly if the first and second genes encode M cone photopigments that differ in their wavelengths of peak sensitivity (Figure 2C).

More rarely, mutations that inactivate one of the first two genes in the array cause red-green color vision deficiency. For recent reviews on common causes of inherited red-green color vision deficiency, see references [8,69]. In addition, exon 3-skipping *OPN1LW* and *OPN1MW* haplotypes have been identified as a cause of red-green color vision deficiency, as described in more detail below.

## 4. Consequences of the LIAVA Haplotype for Vision

Initial studies that identified the LIAVA haplotype as a cause of cone dysfunction aimed to understand specific vision phenotypes: red-green color vision deficiency [24,26,29] and blue cone monochromacy [63]; thus, the initial observations considered only these phenotypes. An early clinical study of individuals with a complex phenotype that included protanopia, moderate to high myopia, astigmatism, reduced cone function, and moderately reduced visual acuity also led to the identification of the LIAVA haplotype as the culprit [71]. However, the haplotype was not initially recognized as disease-causing because it represents a combination of known polymorphisms that are individually benign. The subsequent discovery that the LIAVA haplotype causes complete exon 3-skipping explained the color vision phenotype [29] and provided insight into the mechanism by which this and other haplotypes that cause exon 3-skipping might cause nearsightedness.

### 4.1. The LIAVA Haplotype and Blue Cone Monochromacy

**Figure 3:**
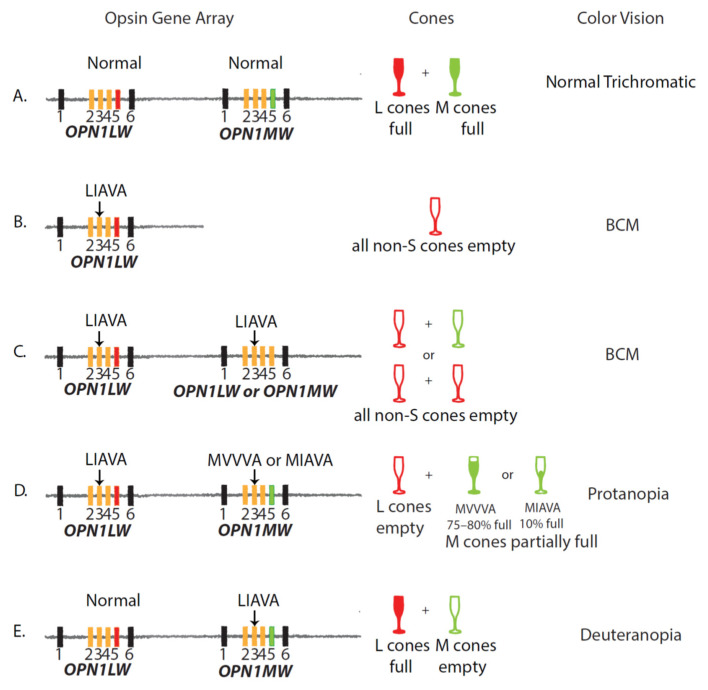
(**A**) Arrays with an *OPN1LW* gene followed by an *OPN1MW* gene, each with haplotypes that splice normally, give rise to L and M cones that have a normal amount of photopigment, which in turn confers normal, trichromatic red-green color vision. Exons are represented by the numbered rectangles, and the color-coding is the same as that described in Figure 2. (**B**) An array with a single *OPN1LW_LIAVA_* gene does not produce any functional photopigment, so all cones except-S cones are devoid of pigment, and a male with this array is an obligate Blue Cone Monochromacy (BCM). (**C**) An array in which the first two genes have the LIAVA haplotype will cause BCM regardless of whether the second gene encodes an *OPN1LW* or *OPN1MW* gene (indicated by a yellow rectangle for exon 5) because all cones except S cones will be devoid of photopigment. (**D**) An array in which the first gene is *OPN1LW_LIAVA_* and the second gene is either *OPN1MW*_MVVVA_ or *OPN1MW_MIAVA_* will cause protanopia in males because the L cones will be devoid of photopigment, and the M cones will have photopigment. Thus, color vision will be mediated by M and S cones. (**E**) An array in which the *OPN1LW* gene has a normal (non-exon 3-skipping) haplotype and the second gene is an *OPN1MW_LIAVA_* will cause deuteranopia in males because only L cones and S cones will contain functional photopigment. Arrays in this figure correspond to those found in patients described in Tables S1–S3 [22,27,31,33,39].

Compared to opsin gene arrays that underlie normal color vision (Figure 3A), arrays in which all expressed opsin genes have the LIAVA haplotype do not produce functional L or M cones. Consequently, males who inherit these arrays are obligate blue cone monochromats. Key features of BCM caused by an LIAVA opsin gene haplotype expressed in all L/M cones include pathological myopia, very poor visual acuity, and a severely reduced or absent cone function measured using the photopic electroretinogram (ERG) (Table S1) [22,27,31]. All of the subjects listed in Table S1 had reduced visual acuity, reduced cone function, and most had pathological myopia.

The fovea is the center of the field of vision and the location of the highest visual acuity. Cone photoreceptors are concentrated in the fovea. In conventional vision charts, a person with normal visual acuity is able to resolve 1 min of arc of visual angle. The size of each letter is such that its strokes will subtend 1 min of arc at a specified distance. In standard notation, visual acuity is as a fraction where the numerator indicates the viewing distance, and the denominator indicates the size of the letter. At 20 feet away, an observer who can just recognize the line on the chart with letters having strokes of 1 min of arc has a visual acuity of 20/20. An observer who requires letters twice that size has an acuity of 20/40, and so forth. Under ideal conditions, an observer with excellent vision can just resolve the fine detail for which the angular subtense approaches that of a single cone [72]. L- and M-cones mediate high-acuity achromatic spatial vision. Blue cone monochromacy is characterized by poor visual acuity because affected males lack functional L and M cones.

After birth, human eyes undergo a controlled axial elongation (termed emmetropization) until the length of the eye matches the power of the optical components (lens and cornea), resulting in high acuity vision. A visual feedback mechanism governs emmetropization, whereby L and M cones play a critical role in detecting and monitoring the pattern of dark and light that characterizes sharply focused versus blurred images. Nearsightedness (myopia) results if the eye grows too long, failing to stop elongating after reaching the optimal length. Presumably, disruption of the signals, initiated by light absorption in L and M cones that guide emmetropization, explains the high myopia associated with the LIAVA opsin gene.

### 4.2. The LIAVA Haplotype and Inherited Red-Green Color Vision Deficiency

Males are obligate dichromats if they have an X-chromosome opsin gene array in which a gene in one of the expressed positions carries the LIAVA haplotype (Figure 3D,E). Males with an *OPN1LW_LIAVA_* gene and an *OPN1MW* gene that is transcribed and translated into functional photopigment will have protanopia because all L cones will be devoid of photopigment. Only S and M cones will contribute to vision (Figure 3D). Likewise, males with an *OPN1LW* gene that is transcribed and translated into functional photopigment but with an *OPN1MW*_LIAVA_ gene will have deuteranopia (Figure 3E) because M cones will be devoid of photopigment. Only S and L cones will contribute to color vision. Males with these array types for whom clinical characteristics were reported in the literature are listed in Table S2.

Results from color vision testing performed under typical clinical conditions are not reliable. Clinical color vision testing is often not performed under valid conditions or with appropriate tests, and the interpretation of the test results is often not performed by a knowledgeable person. In Table S2, color vision is listed as impaired if color vision testing was performed but did not distinguish between protan and deutan defects. In Table S2, members of the MOL0152 family were diagnosed with blue cone monochromacy in the absence of color vision testing. They all have a genotype consistent with protanopia, not blue cone monochromacy. Three other subjects received a clinical diagnosis of blue cone monochromacy (BCM160-23130, ZD314-18057, BCM51-12359), but all three have genotypes consistent with protanopia, not blue cone monochromacy, and color vision tests performed were not adequate to reliably detect BCM. These protanopes with the LIAVA mutation are expected to have many fewer cones than normal, and thus, lower than normal visual acuity, as described more fully below.

X-chromosome opsin gene arrays underlying the common forms of inherited red-green color vision deficiency are not associated with a reduced number of functional cones; thus, individuals with red-green CVD usually have normal acuity [24,73]. Furthermore, even for individuals with a single opsin gene on the X-chromosome that is normally transcribed and translated, the total number of cones and density of cones do not differ from that observed for normal trichromats, presumably because all non-S cones express the available X-chromosome opsin gene [73,74]. However, deuteranopia and protanopia caused by the LIAVA haplotype are associated with a reduced number of functional cones [24], and there is tremendous variability in visual acuity (Table S2).

For males with arrays such as those illustrated in Figure 3D,E in which one of the first two genes has the LIAVA haplotype, and the other has a “normal” haplotype, depending on the fraction of cones that are devoid of photopigment, visual acuity may be reduced to a greater or lesser extent. Variation in the ratio of L: M cones in males with normal color vision reflects the relative number of cones that express the first versus the second opsin gene in the X-chromosome array. Among males with normal color vision, the ratio of L:M cones varies tremendously, with a mean of 2L:1M cones for males of European ancestry [75,76], which corresponds to 67% of L and M cones being L cones. The mean for African or African American males is 1 L for every 1 M cone, or about 50% L cones [77]. A corresponding variation in the relative number of cones expressing the first versus downstream genes in arrays such as those illustrated in Figure 3D,E would correspond to variability in the fraction of empty versus functioning cones, which likely contributes to variability in visual acuity [33].

High-resolution adaptive optics imaging indicates that the degree of myopia as indicated by the eye’s axial length and spherical equivalent refraction is greater when relatively more cones express the LIAVA haplotype [33,39]. For example, two brothers (Table S2, JC_0195 and JC_0196) differ dramatically in the relative fraction of cones devoid of photopigment and the degree of myopia. The brother with the much larger fraction of cones expressing the LIAVA opsin gene (JC_0195) is more severely nearsighted. His visual acuity is also worse than his brother’s (JC_0196). Additionally, subjects MM_0142 and MM_0145 have a much smaller fraction of cones expressing the LIAVA haplotype than subjects JC_0609 and JC_0195. MM_0142 is only slightly myopic where MM_0145 is not myopic and both have much better visual acuity than JC_0609 and JC_0195 (Table S2). Adaptive optics imaging for subject JC_0084, who has normal visual acuity, suggests that about one-third of his cones express the LIAVA opsin gene [24].

Results in Table S2 demonstrate that the LIAVA haplotype is often associated with reduced visual acuity, and such individuals often receive a clinical diagnosis of blue cone monochromacy, even in the absence of definitive color vision test results. Blue cone monochromacy is usually associated with decreased acuity. Thus, poor visual acuity in the individuals with array structures illustrated in Figure 3D or Figure 3E may contribute to their misdiagnosis as BCM rather than red-green color vision deficient with a reduced number of L/M cones.

## 5. The MIAVA Haplotype

The MIAVA haplotype is an exon 3-skipping haplotype, but unlike LIAVA, a small amount of correctly spliced mRNA is made (Table 2). Patients with arrays containing an opsin gene with the MIAVA haplotype in one of the expressed positions have been seen in two scenarios (Table S3). In one scenario, both expressed positions contain genes with the MIAVA haplotype, and both are *OPN1LW* genes. Because the cones expressing the *OPN1LW_MIAVA_* genes will have a small amount of functional photopigment, they will have the spectral sensitivity of L cones. This genotype is consistent with deuteranopia with decreased L/M cone function, not BCM; however, the subjects did not participate in color vision testing. These subjects exhibit degenerative changes associated with myopia [78]. The two individuals (S24 and S25, Table S3) are related and have pathological myopia and moderate to severely reduced cone function [31]. In the second scenario, the array has an *OPN1LW_LIA_*_VA_ gene followed by an *OPN1MW_MIAVA_* gene (Table S3). This genotype is consistent with protanopia with decreased cone function and reduced numbers of cones. However, some of these patients received clinical diagnoses of blue cone monochromacy without having their color vision tested.

Males with an opsin gene with the MIAVA haplotype in both expressed positions have better visual acuity than subjects with LIAVA in the first gene and MIAVA in the second gene (Table S3). This is likely due, at least in part, to all cones containing functional photopigment in the former versus a fraction of cones being devoid of photopigment in the latter. Consistent with their cones lacking or having a reduced amount of photopigment, all subjects in Table S3 no measurable or a severely reduced cone ERG.

## 6. The LVAVA Haplotype

The LVAVA haplotype was initially reported in an individual with red-green color vision deficiency [37] and patients diagnosed with Bornholm Eye Disease [79]. Still, it was recognized as harmful only after the splicing defect was discovered because the haplotype represents a combination of individually benign, normal polymorphisms. Bornholm Eye Disease (BED) is characterized by red-green color vision deficiency, high myopia, and X-linked cone dysfunction [80].

### 6.1. LVAVA and Red-Green Color Vision

**Figure 4:**
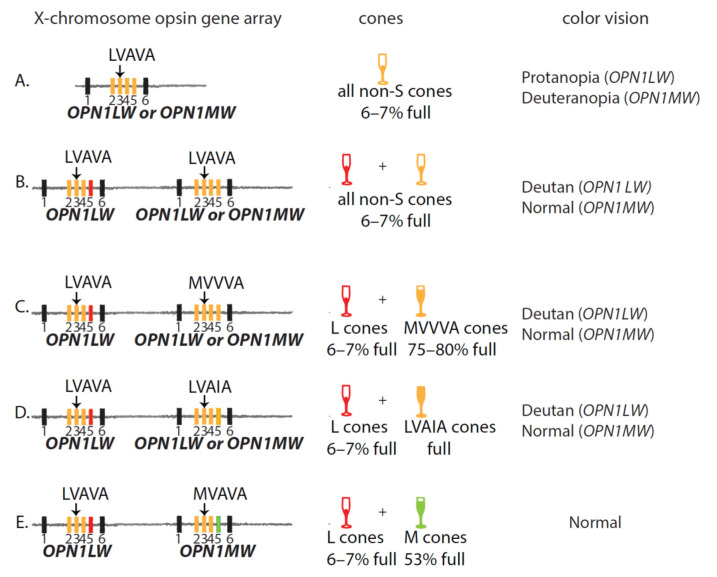
Opsin gene arrays with LVAVA haplotype and the associated color vision. Rectangles represent exons; see Figure 2 and Figure 3 for a description of the color-coding. For example, the yellow rectangle representing exon 5 indicates the gene may be *OPN1LW* or *OPN1MW*. (**A**) Array with a single opsin gene that is either *OPN1LW_LVAVA_* or *OPN1MW_LVAVA_*_._ A male with an array like this will be an obligate deuteranope if the gene is *OPN1LW* or an obligate protanope if the gene is *OPN1MW.* Deuteranopes have functional S and L cones; protanopes have function S and M cones. (**B**) An array in which both expressed positions have the LVAVA haplotype. In a male with this array all non-S cones will have a small amount of functional photopigment. The color vision phenotype depends on whether the second gene is L (deutan) or M (normal). (**C**,**D**) An array with *OPN1LW_LVAVA_* in the first position and an *OPN1LW* or *OPN1MW* gene with the MVVVA (C) or LVAIA (**D**) haplotype in the second position. Males with one of these arrays will have CVD or normal color vision depending on whether the second gene is *OPN1LW* (deutan) or *OPN1MW* (normal). (**E**) An array with *OPN1LW_LVAVA_* and *OPN1MW_MVAVA_*. A male with this array will have functional L and M cones and thus normal color vision. Arrays in this figure are based on patients described in Tables S4–S6 [22,31,32,33,38,39,81].

Unlike the LIAVA haplotype, the splicing defect in the LVAVA haplotype is not expected to cause red-green color vision deficiency because the splicing defect is incomplete. A small amount of correctly spliced mRNA is made and translated into functional photopigment [40]. Thus, the color vision phenotype of males with an LVAVA haplotype will depend on the number of opsin genes in the array and whether the genes in the expressed positions encode opsins of the same or different classes (Figure 4). For example, the initial report of the LVAVA haplotype associated with CVD was in a Japanese male with a single X-chromosome cone opsin gene that was *OPN1MW_LVAVA_*, as illustrated in Figure 4A [37]. The fact that he has a single opsin gene explains his protanopia. A male with *OPN1LW_LVAVA_* genes in both expressed positions (Figure 4B) had deuteranopia (JC_0748, Table S4).

The color vision phenotypes in the BED patients studied by Young et al. [79] are explained by their having opsin gene arrays in which the genes in the expressed positions encode the same class of pigment. For example, affected members of one family had deutan color vision and an array with both expressed opsin genes encoding L opsin (Figure 4C). Affected members of a second family had protan color vision and an array with M opsin genes in both expressed positions (Table S4, JC_0451 and his relatives).

Table S4 lists characteristics of patients who have array structures like those illustrated in Figure 4A–C with an LVAVA haplotype in one of the expressed opsin genes and who have a red-green CVD. Males with a single gene array with an *OPN1LW* gene or an array with *OPN1LW* genes in both expressed positions have deutan color vision deficiency. Males are obligate deuteranopes if the encoded L photopigments have the same spectral sensitivity. They are obligate deuteranomalous if the encoded L photopigments differ in spectral sensitivities or optical densities [60]. For example, the LVAVA and MVVVA photopigments (JC_0758 and JC_0683) have the same peak sensitivity but differ dramatically in optical density due to the greater exon 3-skipping in the LVAVA variant compared to MVVVA. Individuals who behave as anomalous trichromats due to differences in optical density behave as dichromats under bleaching conditions that equalize the optical densities. Thus, the color vision test results depend on the testing conditions.

Some patients were diagnosed with blue cone monochromacy, even though their genotypes are consistent with red-green CVD, not BCM (Table S4, BCM66-16407, BCM112-22852, and BCM112-23518). All three patients have worse visual acuity than is typical of red-green color vision deficiency, which may have contributed to the BCM diagnosis. In addition, affected members of family BCM112 were diagnosed with cone-rod dystrophy characterized by progressive degeneration of both rod and cone photoreceptors. As cones degenerate, color vision may change from red–green CVD to blue cone monochromacy.

Table S5 lists characteristics of patients with arrays comprised of an *OPN1LW_LVAVA_* gene followed by an *OPN1MW* gene with a haplotype that predominately splices correctly. All patients listed have normal trichromatic color vision, as predicted by their genotypes.

### 6.2. The LVAVA Haplotype and Myopia and Visual Acuity

Patients who express an LVAVA opsin gene haplotype in all of their L/M cones tend to have worse visual acuities (highlighted in yellow in Tables S4 and S5) compared to patients who express an LVAVA opsin gene in a fraction of their L/M cones (not highlighted in Tables S4 and S5). The patients highlighted in Table S4 also have reduced cone function. Both the visual acuity and reduced cone function are presumably due to all L/M cones containing a tiny amount of functional photopigment. However, patients in Table S5 over the age of 40 have worse visual acuity than younger patients. These patients are related to each other, so other inherited factors may contribute to the age-related reduction in visual acuity. It may also be due to progressive degeneration in the cones expressing the LVAVA haplotype.

Most of the patients listed in Tables S4 and S5 have pathological myopia. For some, the explanation is that abnormal contrast signals arise between adjacent cones with dramatically different amounts of photopigment (for example, LVAVA vs. MVVVA). For others, the reason may be that abnormal contrast signals arise from adjacent cones that are degenerating at different rates (all non-S cones express LVAVA opsin) [40].

## 7. The MVAVA Haplotype

Like the LVAVA haplotype, the splicing defect in the MVAVA haplotype is not expected to cause red-green color vision deficiency because about 50% of the mRNA is correctly spliced and would give rise to functional photopigment (Table 2). This haplotype has only been observed in *OPN1MW* genes in conjunction with *OPN1LW* genes that specify the LVAVA haplotype [38]. The *OPN1LW*_LVAVA_-*OPN1MW*_MVAVA_ genotype is expected to underlie normal red-green color vision. Patients with this genotype have relatively preserved visual acuity (Table S6) but have severe myopia. The visual acuity and high myopia appear to be similar to what is observed for subjects in Tables S4 and S5 who have *OPN1LW_LVAVA_-OPN1MW_MVVVA_* or *OPN1LW_LVAVA_-OPN1MW_LVAIA._*

## 8. The LIAVS Haplotype

About 20% of the mRNA for the LIAVS haplotype correctly splices exon 3 and gives rise to functional photopigment. Thus, like the MIAVA, MVAVA, and LVAVA haplotypes, the splicing defect in the LIAVS haplotype will not cause color vision deficiency. Nonetheless, all individuals identified thus far with the LIAVS either have a single *OPN1LW* gene with the LIAVS haplotype and no other opsin gene, or they have a second opsin gene that specifies the LIAVA haplotype (Table S7). All of these individuals are obligate deuteranopes because they have only functional S cones and L cones. Due to the reduced amount of functional photopigment in their L cones, they all have reduced cone function as measured by the photopic ERG [27,31]. Two of the three individuals with this haplotype who have been studied were myopic (Table S7), and one was reported as being hyperopic (BCM72-16874, Table S7). However, the hyperopic patient was 71 years old and may have undergone cataract surgery. If so, his hyperopia would be attributable to an intraocular lens implant. The LIAVS haplotype is associated with poor visual acuity, and in subject MOL0250 IV:3, visual acuity worsened with age. The patient with a second opsin gene with the LIAVA haplotype has poorer visual acuity than patients who express the LIAVS haplotype in all L/M cones, presumably due to the non-functional LIAVA cones.

## 9. Mechanisms

### 9.1. Role of Individual SNPs on Exon 3 Splicing

Gardner et al. [31] suggested that the nucleotide difference at c.532 (A/G) is principally responsible for the exon 3-skipping phenotype. However, analysis of the data from Buena-Atienza et al. [22] provides evidence that all of the nucleotide polymorphisms contribute to exon 3-skipping. Moreover, effect size mediated by SNP c.532 depends on the identities of the nucleotides at the other polymorphic positions. Position c.532 is clearly important as LIAIA (Hap 10, Table 2) differs from LIAVA only by the c.532 nucleotide, yet LIAIA splices correctly ~41% of the time compared to 0% for LIAVA (Table 2). However, the MVAVA variant (Hap 9, Table 2) differs from LVAVA (Hap 3) at three nucleotide positions, c.453, c.457, and c.465, and 53% of the MVAVA mRNA is correctly spliced compared to 6.7% for LVAVA, a difference of 46.3%, which is larger than the effect of c.532. These same three SNPs distinguish LIAVA from MIAVA, but MIAVA yields 9–10% correctly spliced mRNA compared to 0 for LIAVA, which is a much smaller effect than observed for these three SNPs in LVAVA vs. MVAVA, suggesting that the nucleotides in codon 171 may modify the contribution of differences at c.453, c.457 and c.465. MIAVA (Hap 5/6, Table 2) differs from MVVVA (Hap 7/8, Table 2) at three positions, c.511 and c.513 in codon 171 and c.521 and MVVVA yields 67 to 70% more correctly spliced mRNA than MIAVA, a much larger effect size than observed for c.532. Finally, LIAVS differs from LIAVA at only one position, c.538, yet LIAVS yields 20% more correctly spliced mRNA than LIAVA.

Comparing incremental increases in exon 3 inclusion from LIAVA (0%) to MIAVA (10.4%) to MVAVA (53%) to MVVVA (80.1%), considering only haplotypes with c.465C (Table 2), suggests the effects are additive. That is, starting with the LIAVA haplotype, changing c.453, c.457 to create MIAVA improves splicing by about 10.4%. Substituting position c.511 and c.513 into MIAVA to create MVAVA increases correct splicing by ~42.6% and altering MVAVA to create MVVVA increases correct splicing by about 27.1%. The sum of the three stepwise changes is 80.1, which is the difference observed between LIAVA and MVVVA. This is also true for substitutions required to go from LIAVS to LIAVA to LIAIA. The c.538 change needed to convert LIAVS to LIAVA reduces the amount of correctly spliced mRNA by about 20.3%. Converting LIAVA to LIAIA increases correct splicing by 40.8%. The difference between LIAVS and LIAIA is 20.5% which is the sum of the effects at c.532 and c.538 individually.

### 9.2. Exon 3 Enhancers and Silencers of Splicing

**Figure 5:**
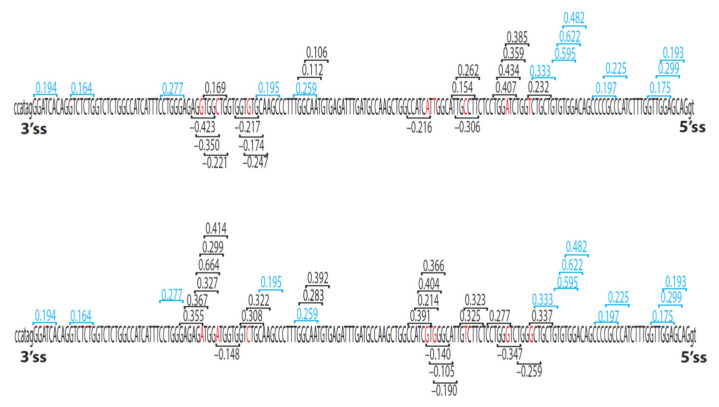
Map showing quantitative effects of hexamers as exonic splicing elements. The upper and lower sequences are for *OPN1LW*/*OPN1MW* exon 3, including the locations of the 5′ and 3′ splice sites (5′ss and 3′ss, respectively). The letters in red font show the common SNPs at each of the eight exon 3 SNPs and, from left to right, correspond to cDNA positions c.453, c.457, c.465, c.511, c.513, c.521, c.528, and c.532. The numbers in blue indicate hexamers that are not affected by the SNPs; the numbers in black change depending on the variant SNPs (upper vs. lower map). Positive values are ESEs; negative values are ESSs. The larger the absolute value of the number, the greater the effect on splicing. The values indicating the impact of each hexamer on splicing are from reference [82].

Ke et al. [82] measured the effects of all possible RNA hexamers on splicing. Figure 5 shows a map of the hexamers identified by Ke et al. as exonic enhancers of splicing (ESEs) or as exonic silencers of splicing (ESEs). Each SNP in exon 3 can abolish an ESE or ESS, create new ESEs or ESSs, or alter the strength of ESEs or ESSs. Thus, the maps in Figure 5 suggest that all exon 3 SNPs potentially contribute to the splicing efficiency of exon 3.

### 9.3. Exon 3 Haplotypes and Disease

Investigations of the effects of the LIAVA, LVAVA, LIAVS, and MIAVA opsin gene haplotypes in humans demonstrate clear evidence of cone dysfunction, in part because it has been possible to evaluate cone function in individuals who express one of these haplotypes in all of their L/M cones [22,24,25,26,27,29,30,32,33,38,39,40,83]. However, this is not true for the MVVVA, MVAVA, or LIAIA haplotypes, and thus their role in vision loss is uncertain. The latter three haplotypes have only been associated with vision disorders in individuals who also expressed one of the four haplotypes listed above, for which there is clear evidence of cone dysfunction (Tables S2–S6). The LIAIA haplotype is quite rare. Only one patient is reported, so whether the disease phenotype observed is due to the LIAIA haplotype is uncertain [22]. The MVVVA haplotype is relatively common and occurs in individuals with no known vision disorders and normally functioning cones [14,21,26]. As noted in Table 2, the MVVVA, MVAVA, and LIAIA haplotypes all exhibit exon 3-skipping. However, how much skipping can be tolerated without causing disease is unknown. Likewise, whether the amount of exon 3-skipping exhibited by the MVVVA, MVAVA, and LIAIA haplotypes contributes to vision loss when expressed in retinas that also express one of the disease-causing haplotypes but not when in retinas that express more normally spliced opsin gene variants is unknown.

## 10. Summary, Conclusions and Future Directions

Exon 3-skipping haplotypes LIAVA, LVAVA, MIAVA, LIAVS are expected to cause cone dysfunction because of the drastically reduced amount or absence of functional photopigment in cones expressing these haplotypes. Data presented in Tables S1–S7, demonstrate that myopia due to excessive axial elongation is associated with these haplotypes. Axial myopia is the primary risk factor for retinal detachment and myopic maculopathy, both of which are degenerative and would further reduce cone function. The risk for these degenerative conditions increases with increasing myopia [78]. Although the retinal and vision phenotypes among patients with these exon 3-skipping haplotypes are often reported to be “non-progressive,” the time frame over which the phenotypes have been monitored is relatively short term. In patients with longer follow up, there is evidence of disease progression. Thus, progression appears to be rather slow in people under the age of 40 years [22,31].

How do exon 3-skipping haplotypes cause severe myopia? We have argued that retinal cone mosaics with adjacent cones differing dramatically in the amount of photopigment signal the presence of constitutive contrast, which stimulates axial elongation of the eye. The contrast signal interferes with the emmetropization process leading to very high degrees of myopia [40]. This “contrast hypothesis” as a cause of myopia led to the development of spectacle lenses designed to reduce contrast, which are currently under evaluation in a multisite clinical trial (NCT03623074). The 12- month data showed that the contrast-reducing spectacle lenses reduced myopia progression by 74% [84], providing support for the contrast hypothesis. Based on the 12-month data, the spectacles have been granted regulatory approval and are authorized for sale as a “myopia control” medical device in the European Union and Canada.

Studies of X-linked high myopia associated with exon 3-skipping opsin gene variants have focused primarily on males, where analyses are simplified because males have one X-chromosome. Some studies have reported female carriers to be asymptomatic [79,81], while others have reported them to be affected [27,32]. As described above, in males, the relative fraction of cones expressing a disease-causing exon 3-skipping haplotype versus a normal opsin gene haplotype influences the severity of myopia and visual acuity loss. More studies are needed to understand the role of exon 3-skipping opsin gene haplotypes in vision in females. Understanding in females is complicated because they have two X-chromosomes. In addition, the exon 3-skipping haplotypes are relatively rare, so the probability that a female will have disease-causing exon 3 haplotypes in opsin genes on both X-chromosomes is very low except in consanguinous families.

The molecular mechanism by which exon 3 SNPs cause exon 3-skipping is an active area of investigation. A better understanding of these mechanisms may provide insight into new therapeutic strategies for associated vision disorders. It remains to be seen whether the contrast-reducing spectacles lenses could reduce myopia if used in young children with LIAVA, LVAVA, MIAVA, or LIAVS opsin gene haplotypes.

## Figures and Tables

**Table 1:** SNPs in *OPN1LW* and *OPN1MW* exon 3.

Codon/Amino Acid Position #	p.151	p.153	p.155	p.171	p.174	p.178	p.180
Amino acids *	R/R	L/M	V/V	I/V	A/V	I/V	S/A
cDNA position **	c.453	c.457	c.465	c.511, c.513	c.521	c.532	c.538
nucleotides	G/A	C/A	G/C	AT/GG	C/T	A/G	T/G

* Single letter amino acid code: T = threonine, I = isoleucine, R = arginine, L = leucine, M = methionine, V = Valine, S = serine, A = alanine. ** cDNA position number 1 is the A of the ATG translational start codon which is codon #1.

**Table 2:** Exon 3-skipping haplotypes associated with vision disorders.

	Nucleotide Differences and Locations in cDNA								Amiino Acid Differences and Codon Number							Correctly Spliced (%)
Haplotype	G/A	C/A	G/C	A/G	T/G	C/T	A/G	T/G	R/R	L/M	V/V	I/V	A/V	I/V	S/A	
	c.453	457	465	511	513	521	532	538	p.151	153	155	171	174	178	180	
Hap 1/2	G	C	G/C	A	T	C	G	G		L		I	A	V	A	0
Hap 3	G	C	G	G	G	C	G	G		L		V	A	V	A	6.7
Hap 4	G	C	G	A	T	C	G	T		L		I	A	V	S	20.3
Hap 5/6	A	A	C/G	A	T	C	G	G		M		I	A	V	A	10.4–8.8
Hap 7/8	A	A	C/G	G	G	T	G	G		M		V	V	V	A	80.1–75.6
Hap 9	A	A	C	G	G	C	A	G		M		V	A	V	A	53.0
Hap 10	G	C	G	A	T	C	A	G		L		I	A	I	A	40.8

## Data Availability

Not applicable.

## Supplementary Materials

The following are available online at https://www.mdpi.com/article/10.3390/genes12081180/s1, Table S1: LIAVA haplotype and arrays that cause blue cone monochromacy, Table S2: LIAVA haplotype and arrays that cause red-green color vision defects, Table S3: MIAVA haplotype and arrays that cause red-green color vision defects, Table S4: LVAVA haplotype and red-green color vision deficiency, Table S5: LVAVA haplotypes and arrays for normal color vision, Table S6: MVAVA haplotype and arrays for normal color vision, Table S7: LIAVS haplotype.

## References

[1] Cartegni L., Chew S.L., Krainer A.R. (2002). Listening to silence and understanding nonsense: Exonic mutations that affect splicing. Nat. Rev. Genet..

[2] Pagani F., Stuani C., Tzetis M., Kanavakis E., Efthymiadou A., Doudounakis S., Casals T., Baralle F.E. (2003). New type of disease causing mutations: The example of the composite exonic regulatory elements of splicing in CFTR exon 12. Hum. Mol. Genet..

[3] Wang G.S., Cooper T.A. (2007). Splicing in disease: Disruption of the splicing code and the decoding machinery. Nat. Rev. Genet..

[4] Ward A.J., Cooper T.A. (2010). The pathobiology of splicing. J. Pathol..

[5] Sterne-Weiler T., Howard J., Mort M., Cooper D.N., Sanford J.R. (2011). Loss of exon identity is a common mechanism of human inherited disease. Genome Res..

[6] Sterne-Weiler T., Sanford J.R. (2014). Exon identity crisis: Disease-causing mutations that disrupt the splicing code. Genome Biol..

[7] Xiong H.Y., Alipanahi B., Lee L.J., Bretschneider H., Merico D., Yuen R.K., Hua Y., Gueroussov S., Najafabadi H.S., Hughes T.R. (2015). RNA splicing. The human splicing code reveals new insights into the genetic determinants of disease. Science.

[8] Neitz J., Neitz M. (2011). The Genetics of Normal and Defective Color Vision. Vis. Res..

[9] Parmley J.L., Urrutia A.O., Potrzebowski L., Kaessmann H., Hurst L.D. (2007). Splicing and the evolution of proteins in mammals. PLoS Biol..

[10] Stergachis A.B., Haugen E., Shafer A., Fu W., Vernot B., Reynolds A., Raubitschek A., Ziegler S., LeProust E.M., Akey J.M. (2013). Exonic transcription factor binding directs codon choice and affects protein evolution. Science.

[11] Ibbotson R.E., Hunt D.M., Bowmaker J.K., Mollon J.D. (1992). Sequence divergence and copy number of the middle- and long-wave photopigment genes in Old World monkeys. Proc. R. Soc. Lond. Ser. B Biol. Sci..

[12] Dulai K.S., Bowmaker J.K., Mollon J.D., Hunt D.M. (1994). Sequence divergence, polymorphism and evolution of the middle-wave and long-wave visual pigment genes of great apes and old world monkeys. Vis. Res..

[13] Hunt D.M., Cowing J.A., Patel R., Appukuttan B., Bowmaker J.K., Mollon J.D. (1995). Sequence and evolution of the blue cone pigment gene in Old and New World primates. Genomics.

[14] Winderickx J., Battisti L., Hibibya Y., Motulsky A.G., Deeb S.S. (1993). Haplotype diversity in the human red and green opsin genes: Evidence for frequent sequence exchange in exon 3. Hum. Mol. Genet..

[15] Verrelli B.C., Tishkoff S.A. (2004). Signatures of selection and gene conversion associated with human color vision variation. Am. J. Hum. Genet..

[16] Nathans J., Thomas D., Hogness D.S. (1986). Molecular genetics of human color vision: The genes encoding blue, green, and red pigments. Science.

[17] Vollrath D., Nathans J., Davis R.W. (1988). Tandem array of human visual pigment genes at Xq28. Science.

[18] Nathans J., Piantanida T.P., Eddy R.L., Shows T.B., Hogness D.S. (1986). Molecular genetics of inherited variation in human color vision. Science.

[19] Chen J.M., Cooper D.N., Chuzhanova N., Férec C., Patrinos G.P. (2007). Gene conversion: Mechanisms, evolution and human disease. Nat. Rev. Genet..

[20] Innan H., Kondrashov F. (2010). The evolution of gene duplications: Classifying and distinguishing between models. Nat. Rev. Genet..

[21] Neitz M., Neitz J., Grishok A. (1995). Polymorphism in the number of genes encoding long-wavelength sensitive cone pigments among males with normal color vision. Vis. Res..

[22] Buena-Atienza E., Ruther K., Baumann B., Bergholz R., Birch D., De Baere E., Dollfus H., Greally M.T., Gustavsson P., Hamel C.P. (2016). De novo intrachromosomal gene conversion from OPN1MW to OPN1LW in the male germline results in Blue Cone Monochromacy. Sci. Rep..

[23] Jacobs G.H. (2009). Evolution of colour vision in mammals. Philos. Trans. R. Soc. Lond. Ser. B Biol. Sci..

[24] Carroll J., Neitz M., Hofer H., Neitz J., Williams D.R. (2004). Functional photoreceptor loss revealed with adaptive optics: An alternate cause of color blindness. Proc. Natl. Acad. Sci. USA.

[25] Crognale M.A., Fry M., Highsmith J., Haegerstrom-Portnoy G., Neitz J., Neitz M., Webster M.A. (2004). Characterization of a novel form of X-linked incomplete achromatopsia. Vis. Neurosci..

[26] Neitz M., Carroll J., Renner A., Knau H., Werner J.S., Neitz J. (2004). Variety of genotypes in males diagnosed as dichromatic on a conventional clinical anomaloscope. Vis. Neurosci..

[27] Mizrahi-Meissonnier L., Merin S., Banin E., Sharon D. (2010). Variable retinal phenotypes caused by mutations in the X-linked photopigment gene array. Investig. Ophthalmol. Vis. Sci..

[28] Carroll J., Dubra A., Gardner J.C., Mizrahi-Meissonnier L., Cooper R.F., Dubis A.M., Nordgren R., Genead M., Connor T.B., Stepien K.E. (2012). The effect of cone opsin mutations on retinal structure and the integrity of the photoreceptor mosaic. Investig. Ophthalmol. Vis. Sci..

[29] Ueyama H., Muraki-Oda S., Yamade S., Tanabe S., Yamashita T., Shichida Y., Ogita H. (2012). Unique haplotype in exon 3 of cone opsin mRNA affects splicing of its precursor, leading to congenital color vision defect. Biochem. Biophys. Res. Commun..

[30] McClements M., Davies W.I., Michaelides M., Young T., Neitz M., Maclaren R.E., Moore A.T., Hunt D.M. (2013). Variations in opsin coding sequences cause X-linked cone dysfunction syndrome with myopia and dichromacy. Investig. Ophthalmol. Vis. Sci..

[31] Gardner J.C., Liew G., Quan Y.H., Ermetal B., Ueyama H., Davidson A.E., Schwarz N., Kanuga N., Chana R., Maher E.R. (2014). Three different cone opsin gene array mutational mechanisms with genotype-phenotype correlation and functional investigation of cone opsin variants. Hum. Mutat..

[32] Li J., Gao B., Guan L., Xiao X., Zhang J., Li S., Jiang H., Jia X., Yang J., Guo X. (2015). Unique Variants in OPN1LW Cause Both Syndromic and Nonsyndromic X-Linked High Myopia Mapped to MYP1. Investig. Ophthalmol. Vis. Sci..

[33] Patterson E.J., Wilk M., Langlo C.S., Kasilian M., Ring M., Hufnagel R.B., Dubis A.M., Tee J.J., Kalitzeos A., Gardner J.C. (2016). Cone Photoreceptor Structure in Patients With X-Linked Cone Dysfunction and Red-Green Color Vision Deficiency. Investig. Ophthalmol. Vis. Sci..

[34] Neitz M., Neitz J., Jacobs G.H. (1991). Spectral tuning of pigments underlying red-green color vision. Science.

[35] Davidoff C., Neitz M., Neitz J. (2016). Genetic Testing as a New Standard for Clinical Diagnosis of Color Vision Deficiencies. Transl. Vis. Sci. Technol..

[36] Neitz M., Neitz J., Cavonius C.R. (1997). Variety of photopigment genes underlying red-green color vision. Colour Vision Deficiencies XIII.

[37] Ueyama H., Kuwayama S., Imai H., Oda S., Nishida Y., Tanabe S., Shichida Y., Yamade S. (2004). Analysis of L-cone/M-cone visual pigment gene arrays in Japanese males with protan color-vision deficiency. Vis. Res..

[38] Orosz O., Rajta I., Vajas A., Takacs L., Csutak A., Fodor M., Kolozsvari B., Resch M., Senyi K., Lesch B. (2017). Myopia and Late-Onset Progressive Cone Dystrophy Associate to LVAVA/MVAVA Exon 3 Interchange Haplotypes of Opsin Genes on Chromosome X. Investig. Ophthalmol. Vis. Sci..

[39] Patterson E.J., Kalitzeos A., Kasilian M., Gardner J.C., Neitz J., Hardcastle A.J., Neitz M., Carroll J., Michaelides M. (2018). Residual Cone Structure in Patients With X-Linked Cone Opsin Mutations. Investig. Ophthalmol. Vis. Sci..

[40] Greenwald S., Kuchenbecker J.A., Rowlan J.S., Neitz J., Neitz M. (2017). Role of a dual splicing and amino acid code in myopia, cone dysfunction and cone dystrophy associated with L/M opsin interchange mutations. Transl. Vis. Sci. Technol..

[41] Lopez-Bigas N., Audit B., Ouzounis C., Parra G., Guigo R. (2005). Are splicing mutations the most frequent cause of hereditary disease?. FEBS Lett..

[42] Dolgin E. (2015). The myopia boom. Nature.

[43] Holden B.A., Fricke T.R., Wilson D.A., Jong M., Naidoo K.S., Sankaridurg P., Wong T.Y., Naduvilath T.J., Resnikoff S. (2016). Global Prevalence of Myopia and High Myopia and Temporal Trends from 2000 through 2050. Ophthalmology.

[44] Holden B., Sankaridurg P., Smith E., Aller T., Jong M., He M. (2014). Myopia, an underrated global challenge to vision: Where the current data takes us on myopia control. Eye.

[45] Holden B.A., Jong M., Davis S., Wilson D., Fricke T., Resnikoff S. (2015). Nearly 1 billion myopes at risk of myopia-related sight-threatening conditions by 2050—Time to act now. Clin. Exp. Optom..

[46] Vitale S., Sperduto R.D., Ferris F.L. (2009). Increased prevalence of myopia in the United States between 1971–1972 and 1999–2004. Arch. Ophthalmol..

[47] Chua S.Y., Sabanayagam C., Cheung Y.B., Chia A., Valenzuela R.K., Tan D., Wong T.Y., Cheng C.Y., Saw S.M. (2016). Age of onset of myopia predicts risk of high myopia in later childhood in myopic Singapore children. Ophthalmic Physiol. Opt..

[48] Holden B.A., Wilson D.A., Jong M., Sankaridurg P., Fricke T.R., Smith Iii E.L., Resnikoff S. (2015). Myopia: A growing global problem with sight-threatening complications. Community Eye Health.

[49] Warner N. (2016). Update on myopia. Curr. Opin. Ophthalmol..

[50] Hammond C.J., Snieder H., Gilbert C.E., Spector T.D. (2001). Genes and Environment in Refractive Error: The Twin Eye Study. Investig. Ophthalmol. Vis. Sci..

[51] Lyhne N., Sjolie A.K., Kyvik K.O., Green A. (2001). The importance of genes and environment for ocular refraction and its determiners: A population based study among 20–45 year old twins. Br. J. Ophthalmol..

[52] Saw S.M. (2003). A synopsis of the prevalence rates and environmental risk factors for myopia. Clin. Exp. Optom..

[53] Wojciechowski R., Congdon N., Bowie H., Munoz B., Gilbert D., West S.K. (2005). Heritability of refractive error and familial aggregation of myopia in an elderly American population. Investig. Ophthalmol. Vis. Sci..

[54] Chen C.Y., Scurrah K.J., Stankovich J., Garoufalis P., Dirani M., Pertile K.K., Richardson A.J., Baird P.N. (2007). Heritability and shared environment estimates for myopia and associated ocular biometric traits: The Genes in Myopia (GEM) family study. Hum. Genet..

[55] Lopes M.C., Andrew T., Carbonaro F., Spector T.D., Hammond C.J. (2009). Estimating heritability and shared environmental effects for refractive error in twin and family studies. Investig. Ophthalmol. Vis. Sci..

[56] Hysi P.G., Wojciechowski R., Rahi J.S., Hammond C.J. (2014). Genome-wide association studies of refractive error and myopia, lessons learned, and implications for the future. Investig. Ophthalmol. Vis. Sci..

[57] Hysi P.G., Mahroo O.A., Cumberland P., Wojciechowski R., Williams K.M., Young T.L., Mackey D.A., Rahi J.S., Hammond C.J. (2014). Common mechanisms underlying refractive error identified in functional analysis of gene lists from genome-wide association study results in 2 European British cohorts. JAMA Ophthalmol..

[58] Neitz J., Neitz M. (2017). Evolution of the circuitry for conscious color vision in primates. Eye.

[59] Schmidt B.P., Sabesan R., Tuten W.S., Neitz J., Roorda A. (2018). Sensations from a single M-cone depend on the activity of surrounding S-cones. Sci. Rep..

[60] Neitz J., Neitz M., He J.C., Shevell S.K. (1999). Trichromatic color vision with only two spectrally distinct photopigments. Nat. Neurosci..

[61] Neitz J., Kuchenbecker J.A., Neitz M. (2020). Ophthalmic Lenses for Treating Myopia. U.S. Patent.

[62] Kuchenbecker J.A., Greenwald S., Neitz M., Neitz J. (2014). Cone-isolating ON-OFF electroretinogram for studying chromatic pathways in the retina. J. Opt. Soc. Am. A.

[63] Nathans J., Davenport C.M., Maumenee I.H., Lewis R.A., Hejtmancik J.F., Litt M., Lovrien E., Weleber R., Bachynski B., Zwas F. (1989). Molecular genetics of human blue cone monochromacy. Science.

[64] Winderickx J., Battisti L., Motulsky A.G., Deeb S.S. (1992). Selective expression of human X chromosome-linked green opsin genes. Proc. Natl. Acad. Sci. USA.

[65] Wang Y., Macke J.P., Merbs S.L., Zack D.J., Klaunberg B., Bennett J., Gearhart J., Nathans J. (1992). A locus control region adjacent to the human red and green visual pigment genes. Neuron.

[66] Wang Y., Smallwood P.M., Cowan M., Blesh D., Lawler A., Nathans J. (1999). Mutually exclusive expression of human red and green visual pigment-reporter transgenes occurs at high frequency in murine cone photoreceptors. Proc. Natl. Acad. Sci. USA.

[67] Deeb S.S., Bisset D., Fu L. (2010). Epigenetic control of expression of the human L- and M- pigment genes. Ophthalmic Physiol. Opt..

[68] Yan W., Peng Y.R., van Zyl T., Regev A., Shekhar K., Juric D., Sanes J.R. (2020). Cell Atlas of The Human Fovea and Peripheral Retina. Sci. Rep..

[69] Neitz M., Patterson S.S., Neitz J. (2019). Photopigment genes, cones, and color update: Disrupting the splicing code causes a diverse array of vision disorders. Curr. Opin. Behav. Sci..

[70] Nathans J., Maumenee I.A., Zrenner E., Sadowski B., Sharpe L.T., Lewis R.A., Hansen E., Rosenberg P., Schwartz M., Heckenlively J.R. (1993). Genetic heterogeneity among blue-cone monochromats. Am. J. Hum. Genet..

[71] Michaelides M., Johnson S., Bradshaw K., Holder G.E., Simunovic M.P., Mollon J.D., Moore A.T., Hunt D.M. (2005). X-linked cone dysfunction syndrome with myopia and protanopia. Ophthalmology.

[72] Neitz M., Green D.G., Neitz J., Albert D.M., Miller J.W. (2008). Section 2: Retina and Vitreous. Albert and Jakobiec’s Principles and Practice of Ophthalmology.

[73] Carroll J., Baraas R.C., Wagner-Schuman M., Rha J., Siebe C.A., Sloan C., Tait D.M., Thompson S., Morgan J.I.W., Neitz J. (2009). Cone photoreceptor mosaic disruption associated with Cys203Arg mutation in the M-cone opsin. Proc. Natl. Acad. Sci. USA.

[74] Wagner-Schuman M., Neitz J., Rha J., Williams D.R., Neitz M., Carroll J. (2010). Color-deficient cone mosaics associated with Xq28 opsin mutations: A stop codon versus gene deletions. Vis. Res..

[75] Carroll J., McMahon C., Neitz M., Neitz J. (2000). Flicker-photometric electroretinogram estimates of L: M cone photoreceptor ratio in men with photopigment spectra derived from genetics. J. Opt. Soc. Am. A.

[76] Hofer H., Carroll J., Neitz J., Neitz M., Williams D.R. (2005). Organization of the human trichromatic cone mosaic. J. Neurosci..

[77] McMahon C., Carroll J., Awua S., Neitz J., Neitz M. (2008). The L:M cone ratio in males of African descent with normal color vision. J. Vis..

[78] Flitcroft D.I. (2012). The complex interactions of retinal, optical and environmental factors in myopia aetiology. Prog. Retin Eye Res..

[79] Young T.L., Deeb S.S., Ronan S.M., Dewan A.T., Alvear A.B., Scavello G.S., Paluru P.C., Brott M.S., Hayashi T., Holleschau A.M. (2004). X-linked high myopia associated with cone dysfunction. Arch. Ophthalmol..

[80] Schwartz M., Haim M., Skarsholm D. (1990). X-linked myopia: Bornholm eye disease. Linkage to DNA markers on the distal part of Xq. Clin. Genet..

[81] Guo X., Xiao X., Li S., Wang P., Jia X., Zhang Q. (2010). Nonsyndromic high myopia in Chinese family mapped to MYP1: Linkage confirmation and phenotypic characterization. Arch. Ophthalmol..

[82] Ke S., Shang S., Kalachikov S.M., Morozova I., Yu L., Russo J.J., Ju J., Chasin L.A. (2011). Quantitative evaluation of all hexamers as exonic splicing elements. Genome Res..

[83] McClements M., Neitz M., Moore A., Hunt D.M. (2010). Bornholm Eye Disease Arises from a Specific Combination of Amino Acid Changes Encoded by Exon 3 of the L/M Cone Opsin Gene. Investig. Ophthalmol. Vis. Sci..

[84] Rappon J., Neitz J., Neitz M., Young G., Chalberg T.W. (2020). CYPRESS 12-Month Results: Safety and Efficacy from a Pivotal Study of Novel Spectacle Lenses Designed to Reduce Myopia Progression.

