# Supplementary material for: Intermixing the OPN1LW and OPN1MW Genes Disrupts the Exonic Splicing Code Causing an Array of Vision Disorders
Source: Genes (Basel). 2021 Jul 29;12(8):1180. doi: 10.3390/genes12081180 (PMC8391646; doi:10.3390/genes12081180)
Supplement: Supplementary file 1 [file genes-12-01180-s001.zip › genes-1310112-supplementary.pdf]

Table S1. LIAVA haplotype and arrays that cause blue cone monochromacy.

**Subject ID**	**Age (yr)**	**Genotype**	**SER OD**	**SER OS**	BCVA
*BCM73-20770	0.25	MLIAVA	-8	-9	20/100
*BCM73-20537	3	MLIAVA	-4	-4	20/100
S16	10	LLIAVA- LLIAVA-MMVVVA	<-6 D	<-6 D	20/60
*MOL0057 III:3	12	MLIAVA	-6	-6	20/160
BCM72-17075	12	LLIAVA-MLIAVA-MMVVVA	0.5	0.5	20/200
*BCM73-17481	14	MLIAVA	-14	-14	20/400
BCM93-19164	14	MLIAVA	-5	-3	20/67
S17	18	LLIAVA- LLIAVA-MMVVVA	<-6 D	<-6 D	20/40
*MOL0057 III:I	20	MLIAVA	-18	-18	20/320
MM_0155	30	LLIAVA - MLIAVA	ND	ND	ND
*BCM73-16953	51	MLIAVA	-12	-12	20/133
S21	73	LLIAVA	<-6 D	<-6 D	20/200

*BCM73-20770, BCM73-20537, BCM73-16953 are relatives, see refernece [1]for pedigree. MOL0057 III:3 and III:1 are relatives, see reference 15 for pedigree. Subjects with IDs starting with BCM are from reference [1], those starting with S are from reference [2], those starting with MOL are from reference [3]. MM_0155 is from reference [4].

Table S2. LIAVA haplotype and arrays that cause red-green color vision defects.

Subject ID	Age (yr)	Color vision	Genotype	SER OD	SER OS	BCVA OD	BCVA OS	Photopic ERG
*MOL0152 III:2	5	ND	L_{LIAVA} - M_{MVVVA}	-5.5	-6.5	20/100	20/200	reduced
*MOL0152 III:3	5	ND	L_{LIAVA} - M_{MVVVA}	-8	-8.75	20/67	20/50	reduced
BCM160-23130	6	impaired	L_{LIAVA} - M_{MVVVA}	-3.75	-5	20/67	20/40	reduced
*MOL0152 III:1	7	ND	L_{LIAVA} - M_{MVVVA}	-9.25	-9.5	20/40	20/50	reduced
ZD314-18057	10	impaired	L_{LIAVA} - M_{MVVVA}	-6.5	-6	20/100	20/100	reduced
JC_0609	11	Protanope	L_{LIAVA} - M_{MVVVA}	-14	-13.75	20/20	20/20	ND
BCM51-12359	12	impaired	L_{LIAVA} - M_{MVVVA}	-14.5	-14.5	20/100	20/100	reduced
MM_0142	13	Protanope	L_{LIAVA} – M_{MVVVA}	-2	-2	ND	ND	ND
*MM_0145	15	Protanope	M_{LIAVA} - M_{MVVVA}	0	0	20/20	20/30	ND
*MM_0144	19	Protanope	M_{LIAVA} - M_{MVVVA}	-8	-8.25	20/70	20/40	ND
JC_0196	28	Protanope	L_{LIAVA}-M_{MVVVA}-M	-6	-5	20/40	20/40	ND
JC_0195	29	Protanope	L_{LIAVA}-M_{MVVVA}-M	-11.5	-12	20/60	20/60	ND
JC_0084	38	Deuteranope	L - M_{LIAVA}	-2.5	-2.5	20/20	20/15	ND

*MOL0512 III:2, III:3, III:1 are relatives; see reference [3] for pedigree; MM_0145 and MM_0144 are brothers, JC_0195 and JC_0196 are brothers, the genotype of MM_0145 not done, presumed to be same as his brother. Subject IDs starting with MOL are from reference [3], those starting with BCM or ZD are from reference [1], those starting with MM or JC are from references [4,5]. ND = not done.

Table S3. MIAVA haplotype and arrays that cause red-green color vision defects.

Subject ID	Age (yr)	Genotype	SER OD	SER OS	BCVA
BCM101-19818	3	L_{LIAVA}-M_{MIAVA}	-6	-6	20/200
*ZD379-19195	7	L_{LIAVA}-M_{MIAVA}	1	1	20/125
*S22	9	L_{LIAVA}- L_{MIAVA}-M_{MIAVA}	<-6 D	<-6 D	20/125
*ZD379-19194	12	L_{LIAVA}-M_{MIAVA}	ND	ND	20/125
*S23	68	L_{LIAVA}- L_{MIAVA}-M_{MIAVA}	>-3D	>-3D	20/200
*S24	34	L_{MIAVA}- L_{MIAVA}-M_{MVVVA}	<-6 D	<-6 D	20/80
*S25	51	L_{MIAVA}- L_{MIAVA}-M_{MVVVA}	<-6 D	<-6 D	20/63

Subjects ZD379-19195, -19194 are relatives; see pedigree in reference [1]. Subjects S22 and S23 are relatives, and S24 and S25 are relatives, see pedigrees in reference [2]. Subjects' IDs starting with BCM or ZD are from reference [1], those beginning with S are from reference[2].

Table S4. LVAVA haplotype and red-green color vision deficiency.

Subject ID	Age Yrs	Color Vision	Diag.	Genotype	AL OD	AL OS	SER OD	SER OS	BCVA	Photopic ERG
BCM194-25474	5	NV	BCM	LLVAVA	ND	ND	-16	-16.25	20/40	reduced
S18	10	I	XLCD	LLVAVA	ND	ND	<-6D	<-6D	20/50	reduced
*JC_11445	26	D	XLCD	LLVAVA – LMVVVA – M – M	25.79	25.65	ND	ND	ND	ND
*JC_0758	33	DA	XLCD	LLVAVA – LMVVVA – M – M	29.18	28.53	-12.75	-10.00	20/50	ND
JC_0347	33	D	XLICD	LLVAVA	24.77	24.25	-3.3	-2	20/200	ND
JC_0683	36	DA	XLCD	LLVAVA – LMVVVA – M – M	27.03	26.70	-8.5	-8.5	20/15	ND
BCM66-16407	41	I	BCM	LLVAVA	ND	ND	-13	-14	20/67	absent
S26	80	I	XLCD	LLVAVA-LLVAVA-MMVVVA	ND	ND	>-3 D	>-3 D	20/125	reduced
*BCM112-22852	6	I	BCM/CRD	MLVAVA	ND	ND	-9	-9.25	20/67	reduced
*JC_0451	12	P	XLCD	MLVAVA – M – M	28.1	27.6	-9.5	-9	20/20	ND
*JC_0448	14	P	XLCD	MLVAVA – M – M	27.75	27.51	-11.5	-10.75	20/25	ND
*BCM112-23518	14	I	BCM/CRD	MLVAVA	ND	ND	-24	-23	20/125	reduced
*JC_0447	17	P	XLCD	MLVAVA – M – M	26.54	26.52	-9.75	-9	20/20	ND
*JC_10340	32	P	XLCD	MLVAVA – MMVVVA – M	28.96	28.08	-17.5	-13	20/20	ND
S19	40	I	XLCD	MLVAVA	ND	ND	<-6D	<-6D	20/80	reduced
JC_0564	45	P	XLICD	MLVAVA	27.08	26.58	-5.8	-4.9	20/100	reduced
S20	49	I	XLCD	MLVAVA	ND	ND	<-6D	<-6D	20/63	reduced

*JC_11445 and JC_11445 are brothers; JC_0683 and JC_0758 are cousins: JC_0451, JC_0448, and JC_0447 are brothers and JC_10340 is their cousin; BCM112-22852 and BCM112-23518 are from the same family. Subject IDs starting with BCM are from reference [1], subject IDs starting with JC_ are from references [4,5], subject IDs starting with S are from reference [2]. CRD=cone-rod dystrophy. Color vision test results: NV = test not valid; D = deuteranope, DA = deuteranomalous, P = protanope, I = impaired. Yellow shaded rows = subjects with LVAVA haplotype expressed in all non-S cones.

Table S5. LVAVA haplotypes and arrays for normal color vision.

Subject ID	Age Yrs	Color Vision	Diag.	Genotype	SER OD	SER OS	BCVA OD	BCVA OS
*BCM133-23364	1	ND	BCM	LLVAVA – MLVAIA	-5	-5	ND	ND
*BCM133-20960	10	I	BCM	LLVAVA – MLVAIA	-5	-6	20/33	20/40
*BCM133-20961	32	ND	BCM	LLVAVA – MLVAIA	-20.75	-19	20/33	20/55
*HM346IV:2	15	Normal	high myopia	LLVAVA – M	-11	-12.25	20/20	20/30
*HM346III:7	27	ND	high myopia	LLVAVA – M	-12.5	-12.5	20/67	20/80
*HM346II:4	47	Normal	high myopia	LLVAVA – M	-13.75	-14	20/200	20/125
*HM346II:2	54	ND	high myopia	LLVAVA – M	-18.25	-16.25	20/200	20/100
*HM295 IV4	6	Normal	high myopia	LLVAVA - M	-10.5	-10.5	ND	ND
*HM295 IV2	12	Normal	high myopia	LLVAVA - M	-8.25	-9	20/30	20/25
*HM295 IV3	12	Normal	high myopia	LLVAVA - M	-9.25	-9.25	20/40	20/40
*HM295 IV7	14	Normal	high myopia	LLVAVA - M	-9.25	-10.25	20/30	20/30
*HM295 IV1	19	Normal	high myopia	LLVAVA - M	-6	-6.25	20/25	20/30
*HM295 IV5	31	Normal	high myopia	LLVAVA - M	-15	-15	20/30	20/40
*HM295 II:5	76	Normal	high myopia	LLVAVA - M	ND	ND	ND	ND
BCM126-20616	41	I	BCM	LLVAVA-MLVAVA	ND	ND	20/80	20/100

*Subject IDs beginning with BCM are from the same reference and BCM133- are from the same family and are from reference [1], subject IDs beginning with HM are from reference [6]. Yellow shaded row = subject expresses LVAVA haplotype in all non-S cones.

Table S6. MVAVA haplotype and arrays for normal color vision.

Subject ID	Age Yrs	Color Vision	genotype	AL OD OS	OD SER	OS SER	BCVA OD	BCVA OS	Photopic ERG
Orosz IV:5	40	ND	LLVAVA – MMVAVA	25.16 25.51	-4.25	-5.25	20/20	20/20	ND
Orosz IV:5	57	DA	LLVAVA – MMVAVA	ND	NA	NA	20/25	20/40	ND
Orosz IV:5	62	I	LLVAVA – MMVAVA	ND	NA	NA	20/25	20/40	absent
Orosz IV:7	51	I	LLVAVA – MMVAVA	27.82 27.57	-5.75	-6.5	20/50	20/33	absent
Orosz V:1	32	ND	LLVAVA – MMVAVA	31.44 30.72	ND	ND	20/20	20/20	ND
Orosz V:1	38	I	LLVAVA – MMVAVA	ND	-20.0	-18.0	20/20	20/20	absent
Orosz V:1	46	I	LLVAVA – MMVAVA	33.11 32.28	-21.0	-19.75	20/20	20/25	ND
Orosz VI:3	42	I	LLVAVA – MMVAVA	31.35 32.49	-22.5	-24.5	20/20	20/20	ND
Orosz VI:6	11	N	LLVAVA – MMVAVA	ND	-6.25	-6.5	20/40	20/20	reduced

All subjects are from the same Hungarian family in reference [7]. Color vision: ND = not done; DA = deuteranomalous, I = impaired, N = normal trichromatic color vision.

Table S7. LIAVS haplotype.

Subject ID	Age (Yr)	Genotype	SER OD	SER OS	BCVA OD	BCVA OS
*MOL0250 III:2	50	L_{LIAVS}	-2.25	-1.75	20/67	20/67
+MOL0250 IV:3	24	L_{LIAVS}	-4.00	-5.50	20/67	20/67
+MOL0250 IV:3	27	L_{LIAVS}	ND	ND	20/67	20/67
+MOL0250 IV:3	32	L_{LIAVS}	-2.75	-5.00	20/80	20/100
MOL0267 III:1	34	L_{LIAVS}	ND	ND	20/67	20/67
BCM72-16874	71	L_{LIAVS}-M_{LIAVA}-M_{MVVVA}	1.75	0.75	20/200	20/200

*members of the same family; see reference [3] for pedigree. +same individual examined over an 8-year period. BCM72-16874 is from reference [1].

References

1. Buena-Atienza, E.; Ruther, K.; Baumann, B.; Bergholz, R.; Birch, D.; De Baere, E.; Dollfus, H.; Greally, M.T.; Gustavsson, P.; Hamel, C.P.; et al. De novo intrachromosomal gene conversion from OPN1MW to OPN1LW in the male germline results in Blue Cone Monochromacy. *Scientific reports* **2016**, *6*, 28253, doi:10.1038/srep28253.
2. Gardner, J.C.; Liew, G.; Quan, Y.H.; Ermetal, B.; Ueyama, H.; Davidson, A.E.; Schwarz, N.; Kanuga, N.; Chana, R.; Maher, E.R.; et al. Three different cone opsin gene array mutational mechanisms with genotype-phenotype correlation and functional investigation of cone opsin variants. *Human mutation* **2014**, *35*, 1354-1362, doi:10.1002/humu.22679.
3. Mizrahi-Meissonnier, L.; Merin, S.; Banin, E.; Sharon, D. Variable retinal phenotypes caused by mutations in the X-linked photopigment gene array. *Investigative Ophthalmology and Visual Science* **2010**, *51*, 3884-3892, doi:doi10.1167/iovs.09-4592
4. Patterson, E.J.; Kalitzeos, A.; Kasilian, M.; Gardner, J.C.; Neitz, J.; Hardcastle, A.J.; Neitz, M.; Carroll, J.; Michaelides, M. Residual Cone Structure in Patients With X-Linked Cone Opsin Mutations. *Invest Ophthalmol Vis Sci* **2018**, *59*, 4238-4248, doi:10.1167/iovs.18-24699.
5. Patterson, E.J.; Wilk, M.; Langlo, C.S.; Kasilian, M.; Ring, M.; Hufnagel, R.B.; Dubis, A.M.; Tee, J.J.; Kalitzeos, A.; Gardner, J.C.; et al. Cone Photoreceptor Structure in Patients With X-Linked Cone Dysfunction and Red-Green Color Vision Deficiency. *Invest Ophthalmol Vis Sci* **2016**, *57*, 3853-3863, doi:10.1167/iovs.16-19608.
6. Li, J.; Gao, B.; Guan, L.; Xiao, X.; Zhang, J.; Li, S.; Jiang, H.; Jia, X.; Yang, J.; Guo, X. Unique variants in OPN1LW cause both syndromic and nonsyndromic X-linked high myopia mapped to MYP1. *Investigative ophthalmology & visual science* **2015**, *56*, 4150-4155.
7. Orosz, O.; Rajta, I.; Vajas, A.; Takacs, L.; Csutak, A.; Fodor, M.; Kolozsvari, B.; Resch, M.; Senyi, K.; Lesch, B.; et al. Myopia and Late-Onset Progressive Cone Dystrophy Associate to LVAVA/MVAVA Exon 3 Interchange Haplotypes of Opsin Genes on Chromosome X. *Invest Ophthalmol Vis Sci* **2017**, *58*, 1834-1842, doi:10.1167/iovs.16-21405.
